# Lethal multiple pterygium syndrome, the extreme end of the *RYR1* spectrum

**DOI:** 10.1186/s12891-016-0947-5

**Published:** 2016-03-01

**Authors:** Ariana Kariminejad, Siavash Ghaderi-Sohi, Hamid Hossein-Nejad Nedai, Vahid Varasteh, Ali-Reza Moslemi, Homa Tajsharghi

**Affiliations:** Kariminejad-Najmabadi Pathology & Genetics Center, Tehran, Iran; Department of Pathology, Shahid Beheshti University of Medical Sciences, Tehran, Iran; Division of Thoracic Surgery, Shahid Beheshti University of Medical Sciences, Tehran, Iran; Department of Pathology, University of Gothenburg, Sahlgrenska University Hospital, SE-413 45 Gothenburg, Sweden; Department of Clinical and Medical Genetics, University of Gothenburg, SE-405 30 Gothenburg, Sweden; Systems Biology Research Centre, School of Biomedicine, University of Skövde, SE-541 28 Skövde, Sweden

**Keywords:** Lethal multiple pterygium syndrome, Akinesia, Arthrogryposis, Foetal hydrops, Cystic hygroma, Ryanodine receptor 1, *RYR1*

## Abstract

**Background:**

Lethal multiple pterygium syndrome (LMPS, OMIM 253290), is a fatal disorder associated with anomalies of the skin, muscles and skeleton. It is characterised by prenatal growth failure with pterygium present in multiple areas and akinesia, leading to muscle weakness and severe arthrogryposis. Foetal hydrops with cystic hygroma develops in affected foetuses with LMPS. This study aimed to uncover the aetiology of LMPS in a family with two affected foetuses.

**Methods and results:**

Whole exome sequencing studies have identified novel compound heterozygous mutations in *RYR1* in two affected foetuses with pterygium, severe arthrogryposis and foetal hydrops with cystic hygroma, characteristic features compatible with LMPS. The result was confirmed by Sanger sequencing and restriction fragment length polymorphism analysis.

**Conclusions:**

*RYR1* encodes the skeletal muscle isoform ryanodine receptor 1, an intracellular calcium channel with a central role in muscle contraction. Mutations in *RYR1* have been associated with congenital myopathies, which form a continuous spectrum of pathological features including a severe variant with onset in utero with fetal akinesia and arthrogryposis. Here, the results indicate that LMPS can be considered as the extreme end of the *RYR1-*related neonatal myopathy spectrum. This further supports the concept that LMPS is a severe disorder associated with defects in the process known as excitation-contraction coupling.

## Background

Autosomal recessive multiple pterygium syndrome (MPS), is a rare but severe disorder associated with anomalies of the skin, muscles and skeleton [1]. There are two forms of MPS, multiple pterygium syndrome Escobar type, (EVMPS, OMIM 26500) the milder form, also referred to as Escobar syndrome, and lethal multiple pterygium syndrome (LMPS, OMIM 253290), the severe form which is fatal before birth or very soon after birth. LMPS has many of the same signs and symptoms as the Escobar type [1]. It is characterised by prenatal growth failure with webbing of the skin (pterygium) present in multiple areas and a lack of muscle movement (akinesia) leading to muscle weakness and severe arthrogryposis. In addition, affected foetuses with LMPS develop foetal hydrops with cystic hygroma [1–3]. LMPS is associated with abnormalities such as hypoplasia of the heart, lung, or brain; intestinal malrotation; kidney abnormalities; cleft palate; and microcephaly. Affected individuals may also develop other anomalies including congenital diaphragmatic hernia, small chest, cryptorchidism, hypoplastic dermal ridges and creases, and more occasionally midforehead haemangioma [1, 4]. Facial anomalies include hypertelorism, epicanthal folds, flat nasal root, microretrognathism and microstomia, down-slanting palpebral fissures, low-set malformed ears, and cleft palate. LMPS is typically fatal in the second or third trimester of pregnancy. It shows phenotypic overlap with the foetal akinesia deformation sequence syndrome (FADS, OMIM 208150) [5, 6].

Mutations in genes encoding components of the neuromuscular junction (NMJ) have been described in FADS and/or LMPS. This includes mutations in the genes *CHRNA1* (OMIM 100690) [7], *CHRND* (OMIM 100720) [7] and *CHRNG* (OMIM 100730) [8], encoding the alpha, delta and gamma subunits of the acetylcholine receptor (AChR), respectively. In addition, mutations in *RAPSN* (OMIM 601592) [3], encoding a postsynaptic protein that connects and stabilizes AChR at the NMJ, *DOK7* (OMIM 610285) [9] and recently identified *MuSK* [10], encoding muscle skeletal receptor tyrosine kinase, have been associated with LMPS and/or FADS.

Here we report the association of the skeletal muscle isoform ryanodine receptor 1, an intracellular calcium channel encoded by *RYR1*, with characteristic LMPS in two foetuses. The foetuses resulted from two pregnancies of non-consanguineous healthy parents of Iranian Caucasian descent. The first pregnancy was terminated at 18 and the second at 13 weeks of gestational age. The results support the concept that LMPS is a severe disorder associated with defects in the process known as excitation-contraction coupling.

## Case 1

**Fig. 1:**
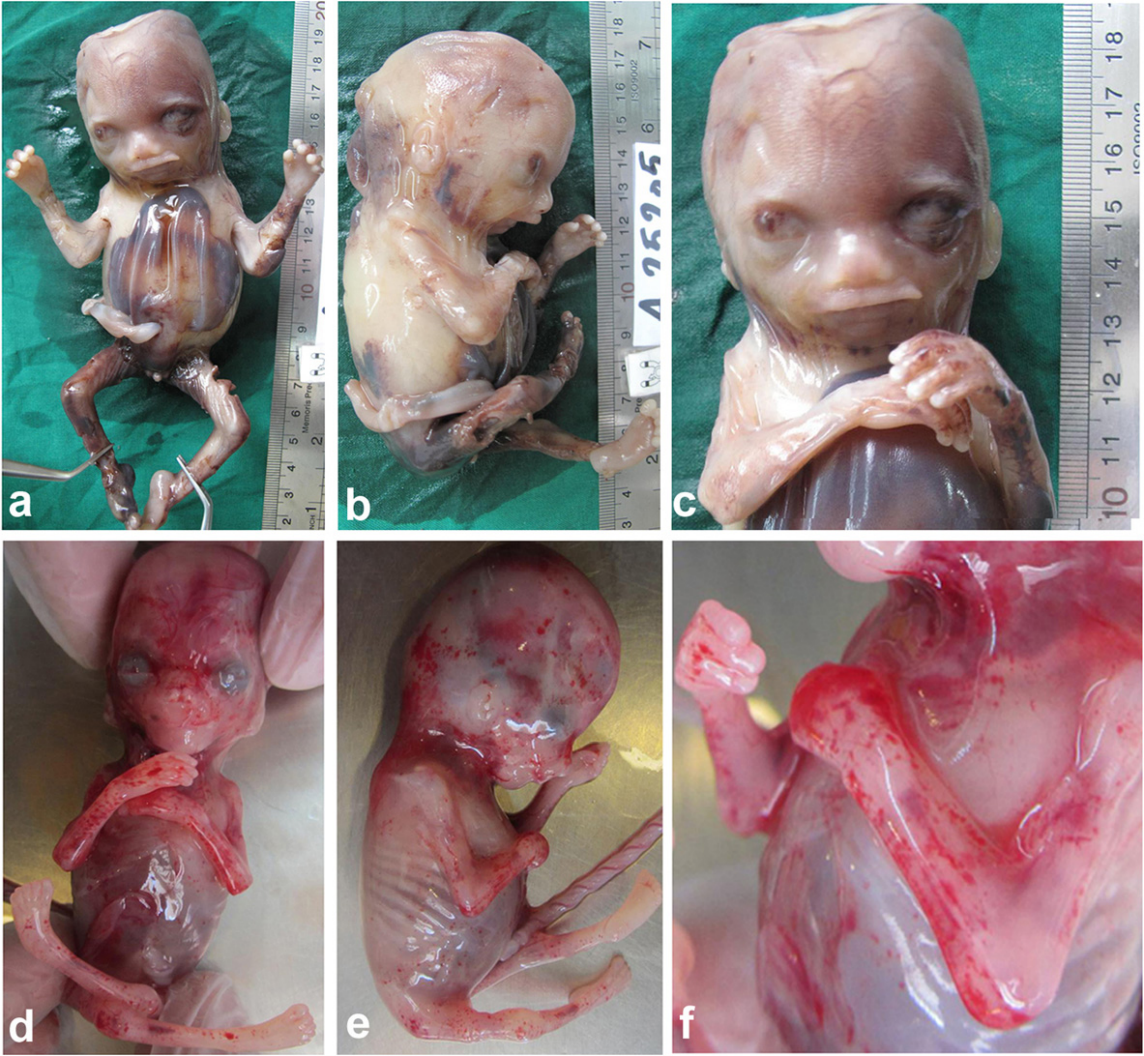
Frontal and lateral view of case 1 and 2. Contracture in elbows and knees are evident in both foetuses (**a, b, d, e**), clubfeet is evident in case 1 (**a**). Note cystic hydromel (**c**). Pterygium is evident in the elbows (**f**)

Case 1 is a female foetus resulted from the first pregnancy of non-consanguineous, healthy, 28-year-old parents of Iranian Caucasian descent. The mother reported reduced foetal movement. Prenatal ultrasound examination at 18 weeks of gestation revealed cystic hygroma, pleural effusion and ascites compatible with hydrops fetalis. The pregnancy was terminated and the foetus and placenta were undertaken for post-mortem and cytogenetic studies. The foetus showed cystic hygroma, contractures in the elbows, hip and knees with pterygium in the elbows (Fig. 1a–c). Two cultures were set up from muscle tissue, which revealed normal 46,XX pattern. DNA was extracted from muscle tissue to perform array Comparative Genomic Hybridisation (aCGH) for the detection of chromosomal aberrations. The results indicated no genomic imbalance exceeding 0.5 MB in size.

## Case 2

Case 2 is the product of the second pregnancy of the couple. Ultra-sound examination at 12 weeks gestational weeks reported cystic hygroma and subcutaneous oedema in the skull thorax and abdomen. Pleural ascites and ascites were not seen. Therapeutic abortion was performed at 13 weeks of gestation. Grossly the second foetus showed cystic hygroma, contractures in the elbows, hip and knees with pterygium in the elbows (Fig. 1d–f). DNA was extracted from foetal tissue for molecular studies.

## Autopsy of case 1

The foetus weighed 135 g (8th centile), was 17,5 (<5th centile) cm crown-heel length and 11.5 cm (<5th centile) crown-rump length. The head, chest and abdomen circumferences were 15, 13.5, and 12 cm, respectively. In head, the fontanels were patent. The eyes were widely set. The ears, nose and mouth were normal. The palate was intact. The chin was small and retracted. The lips and nail beds were normal. The neck showed large cystic hygroma. The thorax and abdomen showed subcutaneous oedema. The extremities were proportioned, each furnished by five fingers or toes. They showed joint contractures predominantly in shoulders, elbows, hips and knees. Webbing of elbows and knees were also seen. Bilateral club-feet was evident. The external genitalia was that of a female foetus. Anus is patent.

The diaphragm, oesophagus, thymus, cardiovascular and respiratory systems, gastrointestinal and genitourinary tracts, liver, spleen, pancreas, internal genitalia, and central nervous system were normal. The serosal cavities contained serosal yellowish effusion. Microscopic examinations of the lung, thymus, liver, kidneys, spleen, bowel wall and pancreas were unremarkable. Unfortunately muscle tissue was not available for microscopy examination at the autopsy.

## Autopsy of case 2

Autopsy was not performed in case 2 due to the small size of the organs.

## Methods

### DNA isolation

Extraction of genomic DNA was performed from whole blood from parents and limb muscle tissue from foetuses, using DNeasy Blood & Tissue kit (Qiagen, Hilden Germany), according to the manufacturer’s instructions. In addition, genomic DNA was extracted from 120 Iranian blood donors, who served as controls.

### Genetic analysis

#### Exome sequence analysis

Whole Exome Sequencing (WES) was performed on DNA from foetuses and their unaffected nonconsanguineous parents. Target enrichment was performed with 3 μg genomic DNA using the Sure SelectXT Human All Exon kit version 4 (Agilent Technologies, Santa Clara, CA, USA) was used to generate barcoded whole-exome sequencing libraries. Libraries were sequenced on the HiSeq2000 platform (Illumina, San Diego, CA, USA) as paired-end 100-bp reads with 30x coverage. Quality assessment of the sequence reads was performed by generating QC statistics with FastQC (http://www.bioinformatics.bbsrc.ac.uk/projects/fastqc). Read alignment to the reference human genome (hg19, UCSC assembly, February 2009) was done using BWA [11] with default parameters. After removal of PCR duplicates (Picard tools, http://picard.sourceforge.net) and file conversion (samtools [12]) quality score recalibration, indel realignment and variant calling were performed with the GATK package [13].

### Variant annotation and selection

Variants were annotated with ANNOVAR [14] using a wide range of databases such as dbSNP build 135, dbNSFP, KEGG, the Gene Ontology project and tracks from the UCSC. A filtering strategy, directed to disease gene candidates, was performed by QIAGEN’s Ingenuity® Variant Analysis™ software (www.qiagen.com/ingenuity) from QIAGEN Redwood City. We focused on exonic variants where the mutation produced a missense change, stop gain or stop loss. We required at least two mutations in the same gene for further analysis. Only those changes that were predicted to be damaging or with unknown impact were kept. We excluded SNPs that were shared with our control dataset (>1 % in dpSNP, the Exome Variant Server (NHLBI), the 1000 Genome Project Database and the human Background Variant DataBase (http://neotek.scilifelab.se/hbvdb/)) as well as those labelled as compound heterozygous.

### Polymerase chain reaction (PCR) and Sanger sequencing

The variants found by whole-exome sequencing in the candidate genes were examined in the individuals by PCR and Sanger sequencing using an ABI 3730XL (GATC Biotech, Constance, Germany and Eurofins MWG Operon, Ebersberg, Germany) if they had a) a variant frequency <1 % in the healthy population or b) a minor allele frequency (MAF) normal MAF in the European population in EVS. Polymerase chain reaction (PCR) was performed on DNA samples from foetuses and their unaffected parents and 120 control individuals. PCR primers are available on request.

### Restriction fragment length polymorphism (RFLP)

DNA samples isolated from 120 Iranian blood donors served as controls. The appearance of the missense and nonsense *RYR1* mutations was evaluated by RFLP analysis in the foetuses and their parents and in the 240 control chromosomes. Loss of a *BsrI* restriction enzyme site was used to assess the *RYR1* mutation c.4567A > C (ACT > CCT), (p.Thr1523Pro) in the foetuses and their parents and in 120 control individuals by digesting a *RYR1* exon 31 PCR product with *Bsr I*. Similarly, loss of a *BccI* restriction enzyme site was used to evaluate the nonsense *RYR1* mutation c.9851G > A (TGG > TAG), (p.Trp3284Ter) in the foetuses and their parents and in 120 control individuals by digesting a *RYR1* exon 66 PCR product with *BccI*.

## Results

### Genetic findings

Data from WES on DNA from foetuses and their parents were analysed through the use of the Ingenuity Variant Analysis (IVA) software (Qiagen, Hilden Germany). The filtering strategy narrowed the starting 255331 variants (17848 genes) to 11 genes. This approach allowed the identification of two novel heterozygous mutation in *RYR1* (Accession: AB209425, GI: 62088429); a novel heterozygous A to C change affecting the highly conserved nucleotide of exon 31 (c.4567A > C) predicted to result in a substitution of the polar uncharged threonine at position 1523 to the nonpolar proline (p.Thr1523Pro), located within the N-terminal, and a novel heterozygous nonsense mutation (c.9851G > A) in exon 66, which changes aromatic nonpolar tryptophan at position 3284 to a stop codon (p.Trp3284Ter), located at the C-terminal. The unaffected father was heterozygous for the c.4567A > C mutation and the c.9851G > A mutation was inherited from the unaffected mother (c.9851G > A). None of the mutations was reported in public databases as a polymorphism. Putative deleterious heterozygous variants in *RYR1* exons 31 and 66, were confirmed in the foetuses and their parents by PCR, RFLP analysis and Sanger sequencing. RFLP analysis excluded the *RYR1* sequence variants in 240 control chromosomes.

## Discussion

Lethal multiple pterygium syndrome is a severe disorder with a heterogeneous range of manifestations associated with anomalies of skin, muscle and skeleton. It is characterised by growth deficiency of prenatal onset with multiple pterygia and flexion contractures as the universal findings, causing severe arthrogryposis, and foetal akinesia. In addition, developmental defects including cardiac hypoplasia, cleft palate, cryptorchidism, intestinal malrotation and microcephaly can be present. In severe cases, subcutaneous oedema may cause foetal hydrops with cystic hygroma and lung hypoplasia [2, 3, 15]. FADS, also known as Pena-Shokeir syndrome type I, closely resemble LMPS. Findings that differentiate LMPS from FADS are fetal hydrops and cystic hygroma. Ptergium is a constant feature of LMPS while it can also be found in some FADS cases.

So far, mutations in the genes encoding proteins involved with neuromuscular transmission are the identified genetic causes of MPS, LMPS and FADS [3, 7, 8]. Moreover, two recent studies revealed association of *RYR1* in four families with FADS/LMPS [16, 17].

Here, we report novel compound missense and nonsense mutations in *RYR1* in two foetuses with autopsy findings compatible with characteristic LMPS. The foetuses resulted from two pregnancies of non-consanguineous healthy parents, which were terminated at 18 and 13 weeks of gestational age, respectively.

Ryanodine receptor 1, encoded by *RYR1*, is the skeletal muscle isoform expressed in the sarcoplasmic reticulum and it functions as a calcium release channel. It is involved in the excitation contraction coupling process and it is composed of two domains: a cytoplasmic domain and a transmembrane pore-forming domain. A group of congenital myopathy and anaesthesia-related malignant hyperthermia are allelic disorders associated with genetic defects in *RYR1* (OMIM 180901) [18, 19]. Congenital myopathies associated with *RYR1* form a continuous spectrum of pathological features that span central core disease, congenital fibre type disproportion, multiminicore disease, and congenital myopathies with prominent nuclear internalisation and large areas of myofibrillar disorganisation initially diagnosed as centronuclear myopathy [18]. A severe central core disease with foetal akinesia has been associated with mutations in *RYR1* [20]. Accordingly, the LMPS can be considered as the ultimate end of phenotypes associated with *RYR1* mutations. Association of LMPS with mutations in genes encoding subunits of the AChR, *RAPS*, encoding a postsynaptic protein that connects and stabilizes AChR at the NMJ, and *RYR1* may indicate that LMPS may be considered as a disease of ion channel.

Apart from congenital myopathy, heterozygous variants in *RYR1* are known cause of susceptibility to malignant hyperthermia, a potentially lethal disorder of skeletal muscle calcium homeostasis [19]. However, there was no history of malignant hyperthermia in either of the parents of the foetuses, probably due to low penetrance of this condition. This can also be explained by the possible influence of several genes on the susceptibility to malignant hyperthermia rather than association of malignant hyperthermia with a major gene defect, as phenotypic and genotypic data are not always concordant. Interestingly, malignant hyperthermia was described as a major complication before death in a brother and a sister with LMPS [21]. It is likely that the LMPS combined with the malignant hyperthermia observed in this sibling was associated with mutation in *RYR1*.

## Conclusion

In conclusion, our result confirms that LMPS is an allelic disorder of anaesthesia-related malignant hyperthermia, a group of congenital myopathies and foetal akinesia syndrome, associated with genetic defects in *RYR1*.

## Ethics approval

The study was reviewed and approved by the local ethical committee, Kariminejad-Najmabadi Pathology & Genetics ethical committee board Tehran, Iran and by Regional etikprövningsnämnden i Göteborg, the ethical review committee in the Gothenburg region, Sweden.

## Patient consent

Written informed consent was obtained from the parents for genetic analysis of their foetuses and themselves and for publication of this Case report and any accompanying images. A copy of the written consent is available for review by the Editor of this journal.

## References

[1] Hall JG (1984). The lethal multiple pterygium syndromes. Am J Med Genet.

[2] Chen H, Immken L, Lachman R, Yang S, Rimoin DL, Rightmire D, Eteson D, Stewart F, Beemer FA, Opitz JM et al. Syndrome of multiple pterygia, camptodactyly, facial anomalies, hypoplastic lungs and heart, cystic hygroma, and skeletal anomalies: delineation of a new entity and review of lethal forms of multiple pterygium syndrome. Am J Med Genet. 1984;17(4):809–26. 10.1002/ajmg.1320170411 6720746

[3] Vogt J, Harrison BJ, Spearman H, Cossins J, Vermeer S, ten Cate LN, Morgan NV, Beeson D, Maher ER. Mutation analysis of CHRNA1, CHRNB1, CHRND, and RAPSN genes in multiple pterygium syndrome/fetal akinesia patients. Am J Hum Genet. 2008;82(1):222–7. 10.1016/j.ajhg.2007.09.016 PMC2253973 18179903

[4] Froster UG, Stallmach T, Wisser J, Hebisch G, Robbiani MB, Huch R, Huch A. Lethal multiple pterygium syndrome: suggestion for a consistent pathological workup and review of reported cases. Am J Med Genet. 1997;68(1):82–5. 10.1002/(sici)1096-8628(19970110)68:1<82::aid-ajmg16>3.0.co;2-k 8986282

[5] Moessinger AC (1983). Fetal akinesia deformation sequence: an animal model. Pediatrics.

[6] Ravenscroft G, Sollis E, Charles AK, North KN, Baynam G, Laing NG (2011). Fetal akinesia: review of the genetics of the neuromuscular causes. J Med Genet.

[7] Michalk A, Stricker S, Becker J, Rupps R, Pantzar T, Miertus J, Botta G, Naretto VG, Janetzki C, Yaqoob N et al. Acetylcholine receptor pathway mutations explain various fetal akinesia deformation sequence disorders. Am J Hum Genet. 2008;82(2):464–76. 10.1016/j.ajhg.2007.11.006 PMC2427255 18252226

[8] Morgan NV, Brueton LA, Cox P, Greally MT, Tolmie J, Pasha S, Aligianis IA, van Bokhoven H, Marton T, Al-Gazali L et al. Mutations in the embryonal subunit of the acetylcholine receptor (CHRNG) cause lethal and Escobar variants of multiple pterygium syndrome. Am J Hum Genet. 2006;79(2):390–5. 10.1086/506256 PMC1559492 16826531

[9] Vogt J, Morgan NV, Marton T, Maxwell S, Harrison BJ, Beeson D, Maher ER. Germline mutation in DOK7 associated with fetal akinesia deformation sequence. J Med Genet. 2009;46(5):338–40. 10.1136/jmg.2008.065425 19261599

[10] Wilbe M, Ekvall S, Eurenius K, Ericson K, Casar-Borota O, Klar J, Dahl N, Ameur A, Anneren G, Bondeson ML. MuSK: a new target for lethal fetal akinesia deformation sequence (FADS). J Med Genet. 2015;52(3):195–202. 10.1136/jmedgenet-2014-102730 25612909

[11] Li H, Durbin R (2009). Fast and accurate short read alignment with Burrows-Wheeler transform. Bioinformatics.

[12] Li H, Handsaker B, Wysoker A, Fennell T, Ruan J, Homer N, Marth G, Abecasis G, Durbin R. The sequence alignment/Map format and SAMtools. Bioinformatics. 2009;25(16):2078–9. 10.1093/bioinformatics/btp352 PMC2723002 19505943

[13] McKenna A, Hanna M, Banks E, Sivachenko A, Cibulskis K, Kernytsky A, Garimella K, Altshuler D, Gabriel S, Daly M et al. The genome analysis toolkit: a MapReduce framework for analyzing next-generation DNA sequencing data. Genome Res. 2010;20(9):1297–303. 10.1101/gr.107524.110 PMC2928508 20644199

[14] Wang K, Li M, Hakonarson H (2010). ANNOVAR: functional annotation of genetic variants from high-throughput sequencing data. Nucleic Acids Res.

[15] Fredfeldt KE, Holm HH, Pedersen JF (1984). Three-dimensional ultrasonic scanning. Acta Radiol Diagn (Stockh).

[16] McKie AB, Al-Saedi A, Vogt J, Stuurman KE, Weiss MM, Shakeel H, Tee L, Morgan NV, Nikkels PG, van Haaften G et al. Germline mutations in RYR1 are associated with foetal akinesia deformation sequence/lethal multiple pterygium syndrome. Acta Neuropathol Commun. 2014;2(1):148. 10.1186/s40478-014-0148-0 PMC4271450 25476234

[17] Ellard S, Kivuva E, Turnpenny P, Stals K, Johnson M, Xie W, Caswell R, Lango Allen H. An exome sequencing strategy to diagnose lethal autosomal recessive disorders. Eur J Hum Genet. 2015;23(3):401–4. 10.1038/ejhg.2014.120 PMC4205099 24961629

[18] Monnier N, Romero NB, Lerale J, Nivoche Y, Qi D, MacLennan DH, Fardeau M, Lunardi J. An autosomal dominant congenital myopathy with cores and rods is associated with a neomutation in the RYR1 gene encoding the skeletal muscle ryanodine receptor. Hum Mol Genet. 2000;9(18):2599–608. 10.1093/hmg/9.18.2599 11063719

[19] Fujii J, Otsu K, Zorzato F, de Leon S, Khanna VK, Weiler JE, O'Brien PJ, MacLennan DH. Identification of a mutation in porcine ryanodine receptor associated with malignant hyperthermia. Science. 1991;253(5018):448–51. 10.1126/science.1862346 1862346

[20] Romero NB, Monnier N, Viollet L, Cortey A, Chevallay M, Leroy JP, Lunardi J, Fardeau M. Dominant and recessive central core disease associated with RYR1 mutations and fetal akinesia. Brain. 2003;126(Pt 11):2341–9. 10.1093/brain/awg244 12937085

[21] Robinson LK, O’Brien NC, Puckett MC, Cox MA (1987). Multiple pterygium syndrome: a case complicated by malignant hyperthermia. Clin Genet.

